# A Modified Duhem Model for Rate-Dependent Hysteresis Behaviors

**DOI:** 10.3390/mi10100680

**Published:** 2019-10-09

**Authors:** Jinqiang Gan, Zhen Mei, Xiaoli Chen, Ye Zhou, Ming-Feng Ge

**Affiliations:** School of Mechanical Engineering and Electronic Information, China University of Geosciences, Wuhan 430074, China; ganjq@cug.edu.cn (J.G.); mz@cug.edu.cn (Z.M.); gysjdd@163.com (X.C.); yezhou72@163.com (Y.Z.)

**Keywords:** piezoelectric ceramic materials, Duhem model, hysteresis model

## Abstract

Hysteresis behaviors are inherent characteristics of piezoelectric ceramic actuators. The classical Duhem model (CDM) as a popular hysteresis model has been widely used, but cannot precisely describe rate-dependent hysteresis behaviors at high-frequency and high-amplitude excitations. To describe such behaviors more precisely, this paper presents a modified Duhem model (MDM) by introducing trigonometric functions based on the analysis of the existing experimental data. The MDM parameters are also identified by using the nonlinear least squares method. Six groups of experiments with different frequencies or amplitudes are conducted to evaluate the MDM performance. The research results demonstrate that the MDM can more precisely characterize the rate-dependent hysteresis behaviors comparing with the CDM at high-frequency and high-amplitude excitations.

## 1. Introduction

Piezoelectric ceramic, a new type of functional material, plays an important role in many real-world applications due to its superior performance in converting electrical energy into mechanical energy [1,2,3,4]. Piezoelectric ceramic actuators (PCAs) are ones of the most important applications of the piezoelectric ceramic material owing to their small size, high accuracy, and fast response. Hysteresis behavior is an inherent characteristic of PCAs and has already become a bottleneck in developing the applications of the PCAs. Therefore, it is of great significance to develop more precise hysteresis models for characterizing hysteresis behaviors.

The existing models of PCAs can be generally divided into the rate-independent and rate-dependent hysteresis models [5]. The rate-independent hysteresis models include the Preisach model [6,7], Prandtle–Ishlinskii model [8,9], Maxwell-slip model [10,11] and polynomial-based hysteresis model [12,13], and can be used to describe the nonlinear relationship between the input voltage and the output displacement of PCAs. However, the input rates of these models are generally lower than that of the rate-dependent hysteresis models, such as the Bouc–Wen model [14,15] and Dahl model [16,17,18]. That is mainly because, unlike the rate-independent hysteresis model, the rate-dependent one can describe the dynamic relationship between the input rate and the output. However, the existing rate-dependent hysteresis models generally have low prediction precision and high complexity of model equations. Therefore, how to construct a new, simpler rate-dependent hysteresis model is an urgent and challenging issue of great significance.

Due to its differential equations, the Duhem model has been used to describe and compensate piezoelectric hysteresis behaviors [19,20,21,22]. For example, C.-J. Lin and P.-T. Lin [23] combined the Bouc–Wen model, Dahl model and Duhem model as a modified Duhem model and presented a feedforward controller. Wang et al. [24] identified the Duhem model by neural network methods and designed a robust adaptive controller to compensate hysteresis behaviors. Xie et al. [25] presented an observer-based adaptive controller based on the Duhem model for piezoelectric actuators. 

The classical Duhem model only characterizes symmetrical hysteresis loops while the actual hysteresis loops of piezoelectric actuators are non-symmetrical. It is worth mentioning the fact that the higher the frequency or the amplitude of the input excitation is, the more serious the hysteresis behaviors are [26,27]. When the frequency or amplitude of input excitation signal is increasing, the non-symmetrical of hysteresis loops is more serious. Therefore, the classical Duhem model already cannot precisely describe rate-dependent hysteresis behaviors at high-frequency and high-amplitude excitations. Thus, Oh and Bernstein [28] proposed the rate-independent and rate-dependent semilinear Duhem models without analyzing the modeling errors in detail at high-frequency and high-amplitude excitations by using a complex model. So far, few efforts have been devoted to developing new hysteresis models based on Duhem model.

Motivated by the aforementioned discussions, this paper proposes a modified Duhem model to describe rate-dependent hysteresis behaviors by introducing trigonometric functions. The proposed model has a simple expression and can detailly characterize rate-dependent hysteresis behaviors precisely at high-frequencies and high-amplitude input excitations. The parameters of models can be easily identified by the nonlinear least squares method. The validity of the proposed model is demonstrated via simulation experiments. The rest of this article is organized as follows: In Section 2, the hysteresis system is constructed to introduce the expression of the classical Duhem model. Section 3 introduces the proposed model and the identification of corresponding parameters. Section 4 aims to verify the validity of the established model, and compares it with the classical Duhem model. The results of the analysis are obtained immediately. The conclusion of this paper is placed in Section 5.

## 2. Classical Duhem Model (CDM)

In 1986, Coleman and Hodgdon [29] proposed a hysteresis model for ferromagnetic materials, which describes the relationship between the magnetic field *H(t)* and magnetic flux *B(t)* as follows:

(1) $$\dot{B}(t)=\alpha \left|\dot{H}(t)\right|\cdot [\beta (H(t))-B(t)]+\gamma \dot{H}(t)$$

where *α*, *β* and *γ* are the parameters controlling the shape and size of the hysteresis loop. According to the relationship between single-input and single-output [28], the hysteresis system is given by

(2) $$\dot{x}(t)=f(x(t),u(t),\dot{u}(t)),x(0)=x_{0},t\geq 0,$$

(3) y(t)=h(x(t),u(t))

where *u(t)* is the input, *y(t)* is the output and *x(t)* is a part of it. When this hysteresis model is used to describe hysteresis system of PCAs, the classical Duhem model (CDM) is proposed and expressed as follows:

(4) $$\left\{ \begin{array}{l} Y(t)=X(t)-h(t)\\ X(t)=k\cdot u(t)\\ \dot{h}(t)=\alpha \dot{u}(t)-\beta \left|\dot{u}(t)\right|h(t)+\gamma \left|\dot{u}(t)\right|u(t) \end{array}\right.$$

where X(t) is the linear component and h(t) is the hysteretic component, u(t) is the input voltage and $\dot{u}\left(t\right)$ is the derivative of voltage, and *k*, *α*, *β* and *γ* are the model parameters.

**Figure 1:**
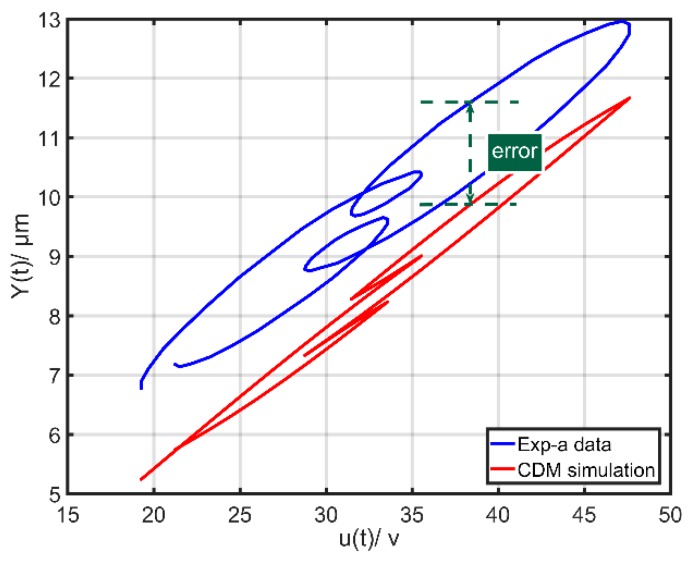
Comparison of the output displacements between experimental data (Exp-a) and simulation of the classical Duhem model (CDM).

**Figure 2:**
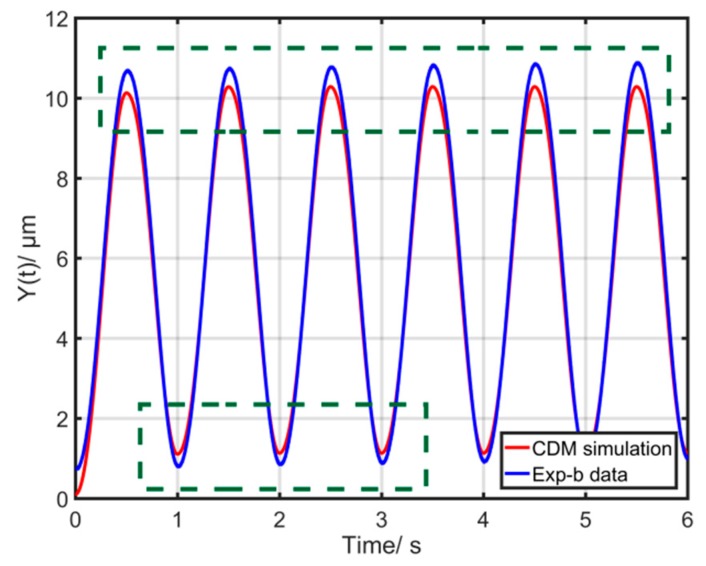
Comparison of the output displacements between experimental data (Exp-b) and simulation of CDM.

To evaluate the performance of the CDM, two groups of experiments were conducted. First of all, a sinusoidal input signal $u_{a}\left(t\right)=\sin\left(2\pi \cdot t\right)+1$ with the frequency of 1 Hz was taken as a reference signal to identify the CDM parameters. The corresponding CDM parameters were identified by utilizing the nonlinear least squares method as *k* = 0.261, *α* = 0.062, *β* = 0.131 and *γ* = 0.001. The first group of experiments, called Exp-a, adopted an input excitation signal $u_{a}\left(t\right)=8\sin\left(2\pi \cdot f_{1}t\right)+6\sin\left(2\pi \cdot f_{2}t\right)+14$ with *f*_1_ = 15 Hz and *f*_2_ = 40 Hz. Figure 1 shows the corresponding comparison between the experimental and simulation results. The results reveal that the error of one point of the CDM (the dotted line) is nearly 2 μm (20% of the displacement range). The maximum modeling error is about 2 μm, which is undoubtedly big. In the second group of experiments, called Exp-b, an input excitation signal $u_{a}\left(t\right)=20\sin\left(2\pi \cdot t\right)+20$ was used. Figure 2 shows the corresponding comparison between the experimental and simulation results of the CDM. The maximum error is about 0.5 μm. It should also be noticed that the point with the maximum modeling error is the point with $\dot{u}(t)\to 0$. These actual experimental results demonstrate that the CDM cannot precisely describe the rate-dependent hysteresis behaviors at high-frequency and high-amplitude excitation signals. 

## 3. Modified Duhem Model (MDM)

The main components of CDM are linear component X(t) and hysteretic component h(t), the former having large influence on the output. Thus, the optimization based on the linear component X(t) is an important research hotspot [30,31]. It should be noted that the main structures of CDM are kept, which can still describe the fundamental characteristics of hysteresis behaviors. With respect to the direct relationship between the input and output, there is an important variation $\dot{u}\left(t\right)$, which has large influences on the whole model when the input frequency is high. The special points $\dot{u}\left(t\right)=0$ are the demarcation points where the input voltage curves go up and down. These important points decide the final shape of hysteresis loops, which has been demonstrated in the previous literature [12,13]. The CDM only characterizes symmetrical hysteresis loops while the actual hysteresis loops of piezoelectric actuators are non-symmetrical. When the frequency or amplitude of input excitation signal is increasing, the non-symmetrical of hysteresis loops is more serious, Therefore, the corresponding errors of the CDM are higher at high-frequency excitations, especially the special points $\dot{u}\left(t\right)=0$. Figure 2 also demonstrates that the points with $\dot{u}(t)\to 0$ have bigger errors. The actual output displacement of PCA varies little when the value of $\dot{u}$ varies greatly at high-frequency excitations. The trigonometric function also has the similar characteristics. Its output varies little when the input varies greatly. Therefore, the trigonometric function as a periodic function has the special points where their derivatives are zero, which can be easily used to compensate the bigger errors of the special points $\dot{u}\left(t\right)=0$. Furthermore, it has a simple expression. Thus, it is a good try to introduce the trigonometric function based on the CDM. Lastly, a modified Duhem model (MDM) based on CDM is proposed and expressed as follows:

(5) $$\left\{ \begin{array}{l} Y(t)=X(t)-h(t)\\ X(t)=k\cdot u(t)+p\cdot u(t)\cdot \cos\left[\left|\dot{u}(t)\right|\right]+q\cdot \dot{u}(t)\\ \dot{h}(t)=\alpha \cdot \dot{u}(t)-\beta \cdot \left|\dot{u}(t)\right|\cdot h(t)+\gamma \cdot u(t)\cdot \left|\dot{u}(t)\right|+\varepsilon \cdot \dot{u}(t)\cdot \sin\left[\left|\dot{u}(t)\right|\right] \end{array}\right.$$

where *p*, *q*, *ε*, *k*, *α*, *β* and *γ* are constants. It must be noticed that when $\dot{u}(t)\to 0$, there is $\cos\left[\left|\dot{u}(t)\right|\right]\to 1$ and $p\cdot u(t)\cdot \cos\left[\left|\dot{u}(t)\right|\right]\to p\cdot u(t)$, which can be perfectly used to compensate for the bigger errors of the special points $\dot{u}(t)\to 0$.

Over the past decade, several methods for parameters identification of models [19,24,32] have been developed, but their identification processes are generally complex. In our previous work [15,33], the nonlinear least squares method is proposed to identify Bouc–Wen model. The nonlinear least squares method adopts the trust-region-reflective algorithm and take the nonlinear least squares function for optimization through the MATLAB/Simulink Optimization Toolbox. Compared with the previous methods, the method is much simpler and can be more easily applied to identify other models. In this paper, the nonlinear least squares method is adopted to identify the MDM and CDM. The objective function F is defined as follows:

(6) $$F=Min\sum_{i=1}^{n}f^{2}(u)$$

(7) $$f(u)=Y_{i}-Y_{i}^{HM}$$

In CDM, there is

(8) $$\left\{ \begin{array}{l} Y_{i}^{HM}(iT)=X(iT)-h(iT)\\ X(iT)=k\cdot u(iT)\\ \dot{h}(iT)=\alpha \cdot \dot{u}(iT)-\beta \cdot \left|\dot{u}(iT)\right|\cdot h(iT)+\gamma \cdot u(iT)\cdot \left|\dot{u}(iT)\right| \end{array}\right.$$

**Figure 3:**
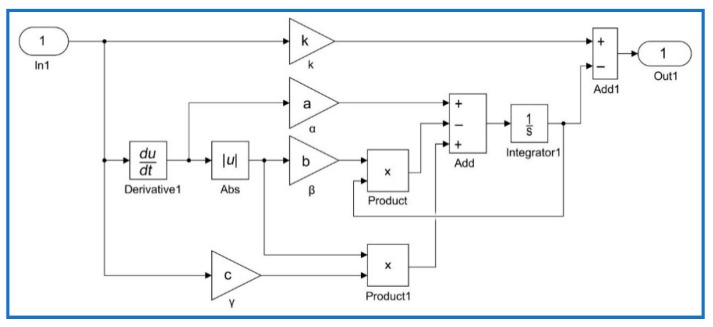
Classical Duhem model implemented with Matlab/Simulink.

**Figure 4:**
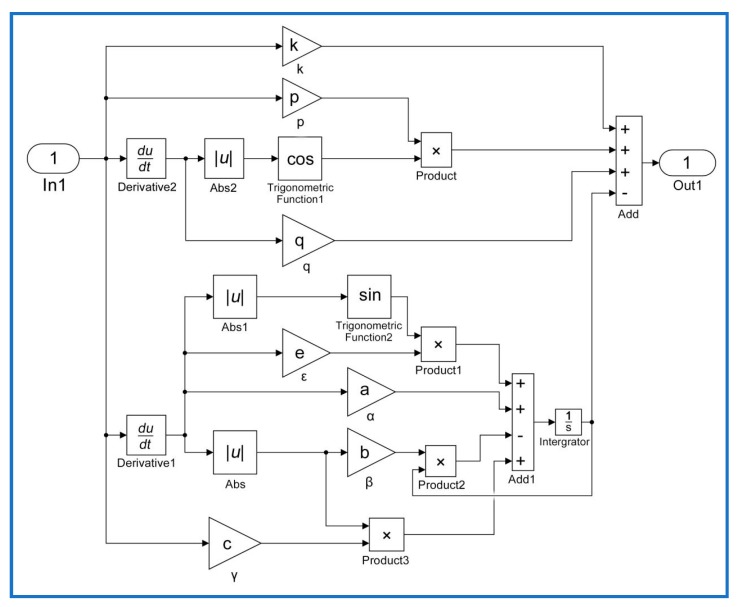
Modified Duhem model implemented with Matlab/Simulink.

In MDM, there is

(9) $$\left\{ \begin{array}{l} Y_{i}^{HM}(iT)=X(iT)-h(iT)\\ X(iT)=k\cdot u(iT)+p\cdot u(iT)\cdot \cos\left[\left|\dot{u}(iT)\right|\right]+q\cdot \dot{u}(iT)\\ \dot{h}(iT)=\alpha \cdot \dot{u}(iT)-\beta \cdot \left|\dot{u}(iT)\right|\cdot h(iT)+\gamma \cdot u(iT)\cdot \left|\dot{u}(iT)\right|+\varepsilon \cdot \dot{u}(iT)\cdot \sin\left[\left|\dot{u}(iT)\right|\right] \end{array}\right.$$

where *i* = 1, 2, 3, ⋯, *n* is the number of sample experiments, *T* is the period of a sample, $Y_{i}$ is the *i*-th output displacement of the PCAs obtained from experiments, and $Y_{i}^{HM}$ is the *i*-th output simulated by the hysteresis model. The corresponding identification steps of the nonlinear least squares method were carried out offline as follows:

(1) Data collection: Experimental data including output displacements and input voltages for piezoelectric actuators were obtained and recorded.
(2) Model implementation: Classical and modified Duhem models were implemented using the MATLAB/Simulink blocks as shown in Figure 3 and Figure 4, respectively. In these figures, block In1 represents the input voltage u(iT) and block Out1 represents the output displacement $Y_{i}^{HM}(iT)$ predicted by the CDM or MDM. Equations (8) and (9) are expressed using MATLAB/Simulink blocks.
(3) Parameter estimation: The trust-region-reflective algorithm was used to identify the parameters of hysteresis models based on experimental data.
(4) Validation: Comparison of the measured and simulation results predicted by hysteresis models were shown, and the corresponding modeling errors were obtained.

## 4. Experimental Results

### 4.1. Experiment Setup

**Figure 5:**
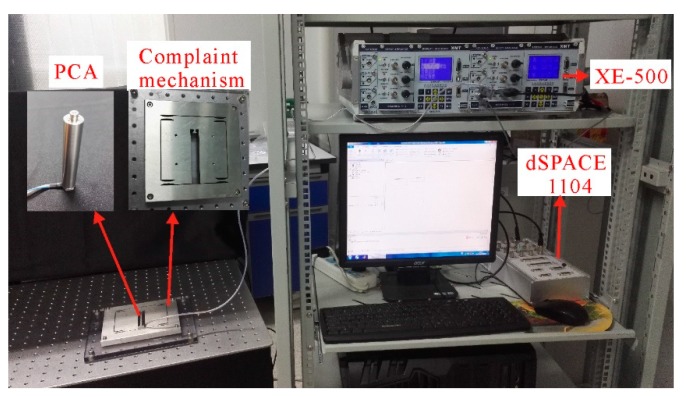
Experimental setup.

To demonstrate the validity of the proposed model, there are some groups of experiments conducted. Figure 5 shows the experimental setup, where a 1-DOF compliant mechanism stage was actuated by a stack piezoelectric ceramic actuator (PST 150/7/60VS12, Coremorrow, Harbin, China). Its nominal displacement was 60 μm for the maximum input voltage of 150 V. This piezoelectric ceramic actuator was made of PZT (Pb-based Lanthanum-doped Zirconate Titanates), whose detailed information is shown in Table 1. The strain gauge position sensor (SGS) included in the piezoelectric ceramic actuator measured the output displacement. dSPACE-DS1104 rapid prototyping controller board equipped with a 16-bit analogue-to-digital converter (ADC) and 16-bit digital-to-analogue converter (DAC) was used to control this 1-DOF compliant mechanism. In addition, an XE-500 controller (Coremorrow, Harbin, China) equipped with an amplifier with 15 times and a signal conditioner was also adopted. A computer with Control Desk 5.0-dSPACE and MATLAB/Simulink was used to conduct all experiments and obtain all experimental data. The detailed experimental steps were carried out as follow:

(1) Building the experimental platform: Connecting all the devices as shown in Figure 5;
(2) Model implementation: Constructing the input excitation signals and designing the control system for the piezoelectric ceramic actuators by using the MATLAB/Simulink blocks;
(3) Experiment start: Actuating piezoelectric ceramic actuators by using control desk 5.0-dSPACE;
(4) Data collection: Obtaining and recording experimental data including output displacements and input voltages for piezoelectric actuators.

### 4.2. Experiment Results and Discussion

In order to evaluate the MDM performance comprehensively, six groups of experiments with different frequencies or amplitudes were conducted. The first and second groups of experiments adopted high-frequency and high-amplitude excitation signals. These two groups had the same amplitudes, but different frequencies. The third and fourth groups adopted low-frequency and low-amplitude excitation signals. The above four groups used multi-frequency signals. The fifth and sixth groups adopted high-frequency and low-amplitude excitation signals. The last two groups used single-frequency signals. The piezoelectric actuator used in our experiments was made of encapsulated stacked piezoelectric ceramics. It must be noted that its input frequency was controlled under 150 Hz to avoid a high dynamic force for security protection. The maximum input voltage of the actuator was 150 V. In the product manuals, it is recommended to control the input voltage within 120 V to guarantee the service life. Therefore, in the experiments, the six groups needed to follow this rule and its input frequencies and amplitudes were generally not high. Finally, the input frequencies including 1, 5, 10, 15, 20, 30, 40 and 50 Hz, and the amplitudes including 3, 5, 6, 8 and 10, were selected randomly following the rule above. In each group of experiments, the chosen excitation signal was used to actuate the PCA in the experimental setup and the experimental output displacements would be recorded and obtained. Subsequently, the displacements predicted by the CDM and MDM were obtained by using the MATLAB/Simulink. Lastly, the comparison results were obtained and drawn.

In the first group of experiments, the excitation signal was $u_{1}\left(t\right)=10\sin\left(2\pi \cdot t\right)+8\sin\left(2\pi \cdot 10t\right)+6\sin\left(2\pi \cdot 20t\right)+24$ with multi-frequency 1, 10 and 20 Hz. The excitation signal in the second group of experiments was $u_{2}\left(t\right)=10\sin\left(2\pi \cdot t\right)+8\sin\left(2\pi \cdot 15t\right)+6\sin\left(2\pi \cdot 40t\right)+24$ with multi-frequency 1, 15 and 40 Hz. The excitation signal in the third group of experiments was $u_{3}\left(t\right)=10\sin\left(2\pi \cdot 5t\right)+6\sin\left(2\pi \cdot 10t\right)+16$ with multi-frequency 5 and 10 Hz. In the fourth group of experiments, the excitation signal was $u_{4}\left(t\right)=5\sin\left(2\pi \cdot t\right)+3\sin\left(2\pi \cdot 5t\right)+8$ with multi-frequency 1 and 5 Hz. The fifth group of experiments took the excitation signal $u_{5}\left(t\right)=5\sin\left(2\pi \cdot 30t\right)+5$ at a frequency of 30 Hz to actuate the piezoelectric actuator. The last group of experiments took another excitation signal $u_{6}\left(t\right)=5\sin\left(2\pi \cdot 50t\right)+5$ at a frequency of 50 Hz. 

To further demonstrate the effectiveness of the MDM, the CDM is set as a comparison. In addition, the modeling errors between the predicted output displacements of the two models and experimental displacements were drawn. In theory, any experimental signals can be used to identify the parameters of the MDM using the nonlinear least squares method. To get better prediction performances of hysteresis models including both the CDM and MDM, the more complex signals were generally adopted for identification, which is acceptable. In this work, the signals of both the first and second group of experiments, which were more complex than the others, were adopted to identify the parameters of the MDM and CDM. The detailed identified parameters of MDM and CDM are shown in Table 2.

**Figure 6:**
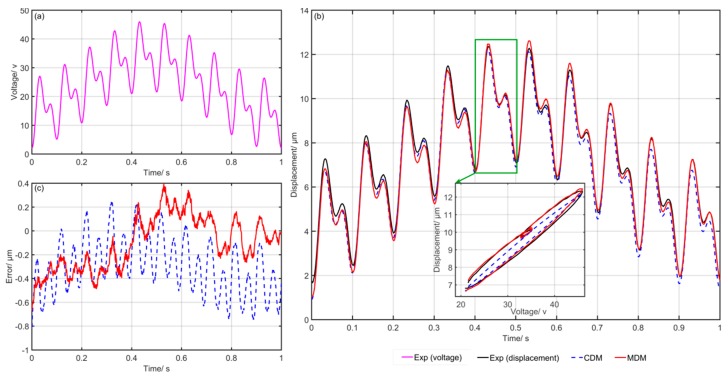
Exp1: Comparison of the experimental and simulation results of the CDM and MDM under $u_{1}\left(t\right)=10\sin\left(2\pi \cdot t\right)+8\sin\left(2\pi \cdot 10t\right)+6\sin\left(2\pi \cdot 20t\right)+24$: (**a**) Time histories of input voltage, (**b**) time histories of output displacements and (**c**) time histories of errors of the CDM and MDM.

**Figure 7:**
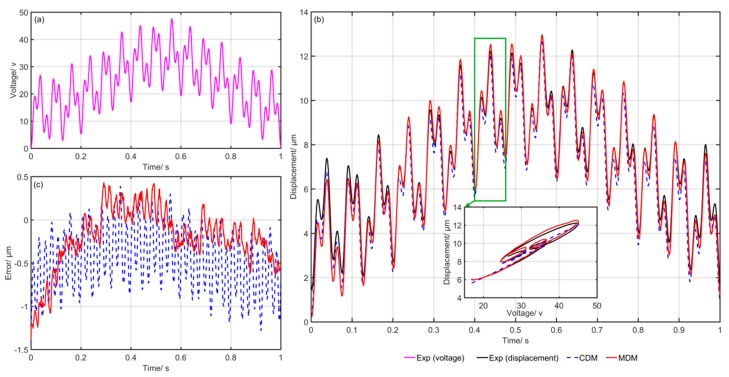
Exp2: Comparison of the experimental and simulation results of the CDM and MDM under $u_{2}\left(t\right)=10\sin\left(2\pi \cdot t\right)+8\sin\left(2\pi \cdot 15t\right)+6\sin\left(2\pi \cdot 40t\right)+24$: (**a**) Time histories of input voltage, (**b**) time histories of output displacements and (**c**) time histories of errors of the CDM and MDM.

**Figure 8:**
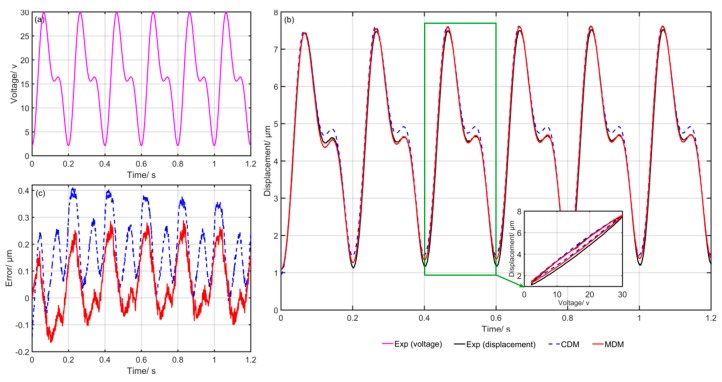
Exp3: Comparison of the experimental and simulation results of the CDM and MDM under $u_{3}\left(t\right)=10\sin\left(2\pi \cdot 5t\right)+6\sin\left(2\pi \cdot 10t\right)+16$: (**a**) Time histories of input voltage, (**b**) time histories of output displacements and (**c**) time histories of errors of the CDM and MDM.

**Figure 9:**
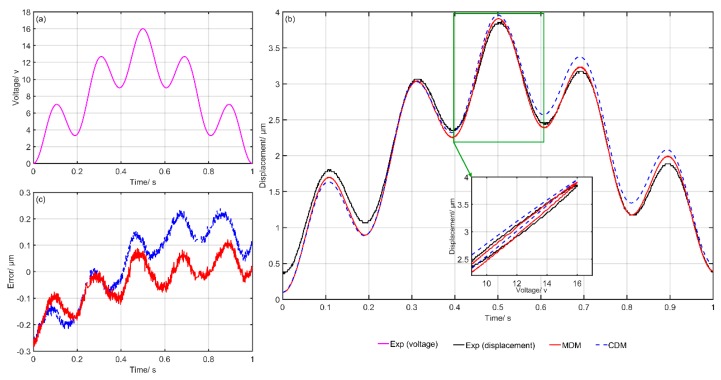
Exp4: Comparison of the experimental and simulation results of the CDM and MDM under $u_{4}\left(t\right)=5\sin\left(2\pi \cdot t\right)+3\sin\left(2\pi \cdot 5t\right)+8$: (**a**) Time histories of input voltage, (**b**) time histories of output displacements and (**c**) time histories of errors of the CDM and MDM.

**Figure 10:**
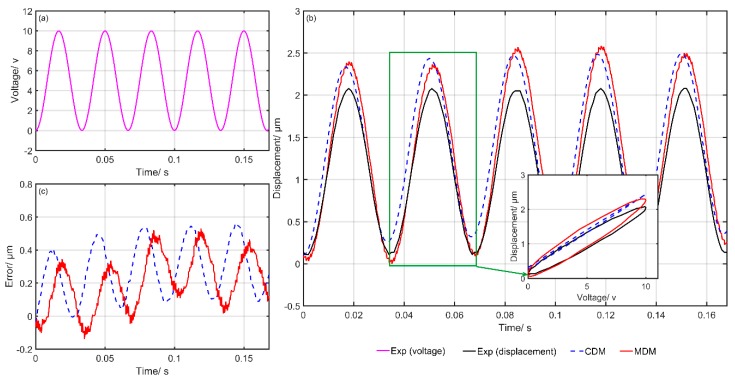
Exp5: Comparison of the experimental and simulation results of the CDM and MDM under $u_{5}\left(t\right)=5\sin\left(2\pi \cdot 30t\right)+5$: (**a**) Time histories of input voltage, (**b**) time histories of output displacements and (**c**) time histories of errors of the CDM and MDM.

**Figure 11:**
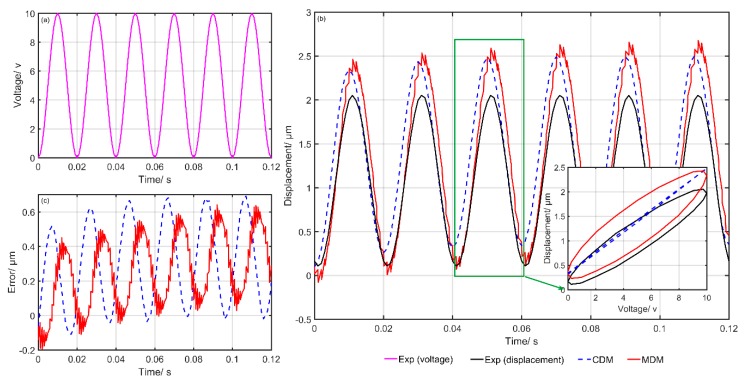
Exp6: Comparison of the experimental and simulation results of the CDM and MDM under $u_{5}\left(t\right)=5\sin\left(2\pi \cdot 50t\right)+5$: (**a**) Time histories of input voltage, (**b**) time histories of output displacements and (**c**) time histories of errors of the CDM and MDM.

Figure 6 shows the comparison of the experimental and simulation results of the CDM and MDM. Figure 6a gives the input voltage at each moment, Figure 6b shows the simulation and experimental results and Figure 6c presents the final modeling errors of the CDM and MDM. The blue dotted line represents the predicted results of the CDM. Meanwhile, the red solid line represents the predicted results of the MDM. It can clearly be seen that the simulation results of the MDM are closer to the experimental data. The modeling errors of the MDM are obviously smaller than that of the CDM. Figure 7, Figure 8, Figure 9, Figure 10 and Figure 11 show the experimental results of the last five groups of experiments. These results further reveal that the MDM simulation results are much closer to the experimental output displacement than the CDM simulation results. The corresponding modeling errors of MDM are much smaller than that of the CDM. In addition, it should be also noted that though the second group of experiments has higher frequency and higher amplitude compared with the other three groups, the MDM still maintains better stability and accuracy compared with the CDM.

To evaluate further the modeling performance of the MDM, the root mean square error $E_{rms}$, the relative root mean square error ξ and optimization ratio φ between the CDM and MDM were employed in comparing the errors of two models as follows:

(10) $$E_{rms}=\sqrt{\frac{{\sum_{i=1}^{n}\left[Y_{\exp}(i)-Y_{pre}(i)\right]}^{2}}{n}}$$

(11) $$\xi =\frac{E_{rms}}{\max\left[Y_{\exp}(i)\right]}\times 100\%$$

(12) $$\varphi =\frac{\left|E_{rms}^{CDM}-E_{rms}^{MDM}\right|}{E_{rms}^{CDM}}\times 100\%$$

where *n* is the total number of the sample and *i* is the *i*-th value in the sample, $Y_{exp}$ is measured from experiments, $Y_{pre}$ represents the displacements predicted by the hysteresis models, and $E_{rms}^{CDM}$ and $E_{rms}^{MDM}$ represent the root mean square error of the CDM and MDM, respectively. The details modeling errors are shown in Table 3.

It can be seen from Table 3 that in the fifth group of experiments (Exp5) at the single-frequency of 30 Hz, $E_{rms}$ and ξ of the MDM were 0.2899 μm and 13.95%,respectively, while those of the CDM were 0.3313 μm and 15.94%, respectively. Compared with the CDM, the MDM can predict more precisely the output displacements and the optimized ratio was 12.50%. In the last group (Exp6) at the single-frequency of 50 Hz, the optimized ratio was up to 14.04%. In the fourth group of experiments (Exp4) with low frequency and amplitude, $E_{rms}$ and ξ of the MDM were 0.0916 μm and 1.2% respectively, while those of the CDM were 0.1426 μm and 3.6%, respectively. Compared with the CDM, the optimized ratio was 35.76%. With the increasing of frequency and amplitude, $E_{rms}$ and ξ of the CDM in the third group of experiments (Exp3) increased to 0.1181 μm and 1.56%, respectively. The corresponding optimization ratio was up to 45.2%. Compared with the two groups above, the first and second groups of experiments (Exp1 and Exp2) had higher amplitude and frequency. The optimization ratios of Exp1 and Exp2 were 34.89% and 31.43%, respectively. Compared with the other three groups, the second group of experiments (Exp2) had the biggest amplitude and frequency, whose $E_{rms}$ and ξ of MDM were 0.5845 μm and 4.6%, respectively. It was found that the modeling errors of both the CDM and MDM increase with the increasing of frequency and amplitude.

These experimental and simulation results clearly reveal that the MDM can describe more precisely rate-dependent hysteresis behaviors at high-frequency and high-amplitude excitations compared with the CDM. 

## 5. Conclusions

In this paper, a modified Duhem model (MDM) is proposed to describe rate-dependent hysteresis behaviors at high-frequency and high-amplitude excitations. The MDM combines trigonometric functions and derivatives of input signal based on the classical Duhem model (CDM). The MDM parameters can be identified easily by the nonlinear least squares method. Six groups of experiments were conducted and the experimental and simulation results show that the MDM can more precisely describe rate-dependent hysteresis behaviors at high-frequency and high-amplitude excitations than the CDM. It is demonstrated that the MDM is effective and useful.

## Figures and Tables

**Table 1:** Information about the piezoelectric ceramics actuator (PCA).

Material	PZT
Nominal stroke (μm) ±15%	60
Stiffness (N/μm) ±20%	15
Length (mm) ±0.3	64
Nominal thrust/tension (N)	1800/300
Electrical capacitance (μF) ±20%	5.4
Resonant frequency (kHz)	15
Stiffness (N/μm) ±20%	15

**Table 2:** Identified parameters of CDM and modified Duhem model (MDM).

Parameters	CDM	MDM
k	0.39854	0.46992
α	0.18695	0.24599
β	0.049939	0.016074
γ	0.0056835	0.0027751
ε	\	−0.030389
p	\	−0.00072364
q	\	−0.00035258

**Table 3:** The simulation errors of the CDM and MDM.

Experiment	CDM		MDM		Optimization Ratio
	$E_{rms}\;(\mu m)$	ξ	$E_{rms}\;(\mu m)$	ξ	φ (%)
Exp1: $u_{1}(t)$	0.3789	3.1	0.2467	1.96	34.89
Exp2: $u_{2}(t)$	0.5845	4.6	0.4008	3.1	31.43
Exp3: $u_{3}(t)$	0.2166	2.84	0.1187	1.56	45.20
Exp4: $u_{4}(t)$	0.1426	3.6	0.0916	1.2	35.76
Exp5: $u_{5}(t)$	0.3313	15.94	0.2899	13.95	12.50
Exp6: $u_{6}(t)$	0.3868	18.86	0.3325	16.21	14.04
